# Acceptability of an mHealth Family Self-management Intervention (myFAMI) for Pediatric Transplantation Families: Qualitative Focus

**DOI:** 10.2196/39263

**Published:** 2022-07-15

**Authors:** Stacee Marie Lerret, Erin Flynn, Rosemary White-Traut, Estella Alonso, Alisha M Mavis, M Kyle Jensen, Caitlin G Peterson, Rachel Schiffman

**Affiliations:** 1 Division of Pediatric Gastroenterology, Hepatology, and Nutrition Department of Pediatrics Medical College of Wisconsin Milwaukee, WI United States; 2 Cincinnati Children’s Hospital Medical Center Cincinnati, OH United States; 3 Department of Nursing Research Children's Wisconsin Milwaukee, WI United States; 4 Division of Pediatric Gastroenterology, Hepatology, and Nutrition Ann & Robert H Lurie Children's Hospital Northwestern University Feinberg School of Medicine Chicago, IL United States; 5 Division of Gastroenterology, Hepatology, and Nutrition Department of Pediatrics Duke University School of Medicine Raleigh, NC United States; 6 Division of Pediatric Gastroenterology Department of Pediatrics University of Utah Salt Lake City, UT United States; 7 Division of Nephrology Department of Pediatrics University of Utah Health Salt Lake City, UT United States; 8 College of Nursing University of Wisconsin-Milwaukee Milwaukee, WI United States

**Keywords:** mHealth, pediatric patients, transplantation, family self-management, qualitative

## Abstract

**Background:**

Around 1800 pediatric transplantations were performed in 2021, which is approximately 5% of the annual rate of solid organ transplantations carried out in the United States. Effective family self-management in the transition from hospital to home-based recovery promotes successful outcomes of transplantation. The use of mHealth to deliver self-management interventions is a strategy that can be used to support family self-management for transplantation recipients and their families.

**Objective:**

The study aims to evaluate the acceptability of an mHealth intervention (myFAMI) that combined use of a smartphone app with triggered nurse communication with family members of pediatric transplantation recipients.

**Methods:**

This is a secondary analysis of qualitative data from family members who received the myFAMI intervention within a larger randomized controlled trial. Eligible participants used the app in the 30-day time frame after discharge and participated in a 30-day postdischarge telephone interview. Content analysis was used to generate themes.

**Results:**

A total of 4 key themes were identified: (1) general acceptance, (2) positive interactions, (3) home management after hospital discharge, and (4) opportunities for improvement.

**Conclusions:**

Acceptability of the intervention was high. Family members rated the smartphone application as easy to use. myFAMI allowed the opportunity for families to feel connected to and engage with the medical team while in their home environment. Family members valued and appreciated ongoing support and education specifically in this first 30 days after their child’s hospital discharge and many felt it contributed positively to the management of their child’s medical needs at home. Family members provided recommendations for future refinement of the app and some suggested that a longer follow-up period would be beneficial. The development and refinement of mHealth care delivery strategies hold potential for improving outcomes for solid organ transplantation patients and their families and as a model to consider in other chronic illness populations.

**Trial Registration:**

ClinicalTrials.gov NCT03533049; https://clinicaltrials.gov/ct2/show/NCT03533049

## Introduction

### Background

Around 1800 pediatric transplantations were performed in 2021, which is approximately 5% of the annual rate of solid organ transplantations carried out in the United States [1]. With successes in achieving high patient and graft survival for pediatric transplantation recipients, focused efforts and the related metrics of quality of care have shifted toward psychosocial patient outcomes. Measures of family experience and outcomes are notably absent [2]. Effective family self-management, the ability and processes used by families to purposefully incorporate health-related behaviors into the family’s daily functioning to prevent or attenuate illness or facilitate the management of complex health regimens, is a key consideration for posttransplantation outcomes [3-5].

Successful outcomes of transplantation require effective family self-management [5,6]. The first 30 days following hospital discharge is a critical time for families to self-manage the additional stressors associated with posttransplantation care including managing the child’s medical schedule (laboratory/clinic appointments, medication administration), impact on family life, and worry about transplantation complications [7-10].

The use of mobile health (mHealth) technology, particularly to deliver self-management interventions, is a strategy that can be used to support family self-management for transplantation recipients [11,12]. Within an mHealth intervention, including monitoring health and adhering to the medical regimen, self-management strategies improved for adult lung transplantation patients [13]. Furthermore, transplantation recipients have reported that they are largely in favor of utilizing mHealth interventions to aid in their recovery [12,14].

The innovative use of mHealth emphasizes the importance of an interactive partnership between families and nurses [12], an essential component of surveillance and care coordination for the transition from hospital to home in complex patient populations [15-20]. The use of mHealth technology in the postdischarge period allows for ease of access by an additional means of communication between families and the medical team, offering the opportunity for outreach and proactive intervention. mHealth technology offers a low-cost and efficient strategy to provide focused health-related messaging [21-23] and reciprocal communication between the nurse and family. The ability to identify factors associated with difficulty managing the child’s illness provides an opportunity to develop effective individualized family-centered interventions that have significant implications for care decisions, complications, and health care use.

Our team has previously conducted a pilot study of the implementation of a family self-management intervention (*myFAMI*) for families of pediatric heart, kidney, or liver transplantation recipients [24]. The effectiveness of interventions is tied to successful implementation [25]. Our initial evaluation of the intervention included quantitative assessments of feasibility, acceptability, and efficacy [26]. Family member use of the app was feasible in our study as 100% (21/21) of primary family members completed the app at least one day after discharge. More specifically, 81% (17/21) of primary family members used the app at a high frequency by completing the app at least 24/30 days after hospital discharge. Acceptability was evident with high nurse response rate (99%, 133/134) to family member trigger alerts within 2 hours. Improvements in patient outcomes of postdischarge coping, family quality of life, self-efficacy, and utilization of health care resources were in the expected direction [24]. The purpose of this analysis was to explore acceptability of myFAMI through an in-depth analysis of family member experiences with the myFAMI intervention. This exploration provides information for improving the intervention for use in future studies with pediatric transplantation families.

### myFAMI App Description

**Figure 1:**
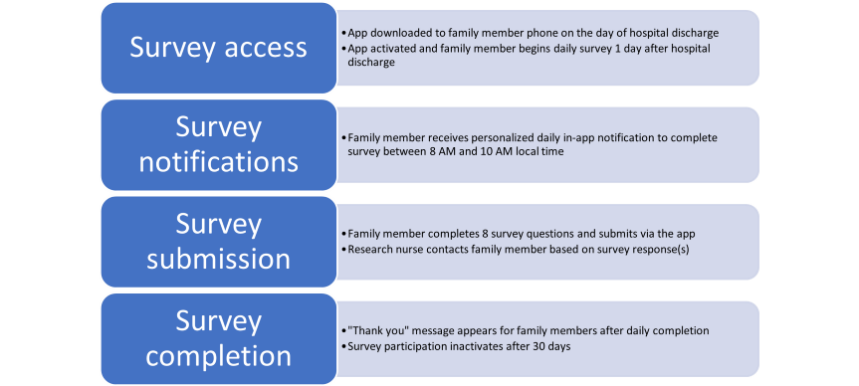
Summary of the myFAMI app workflow.

**Figure 2:**
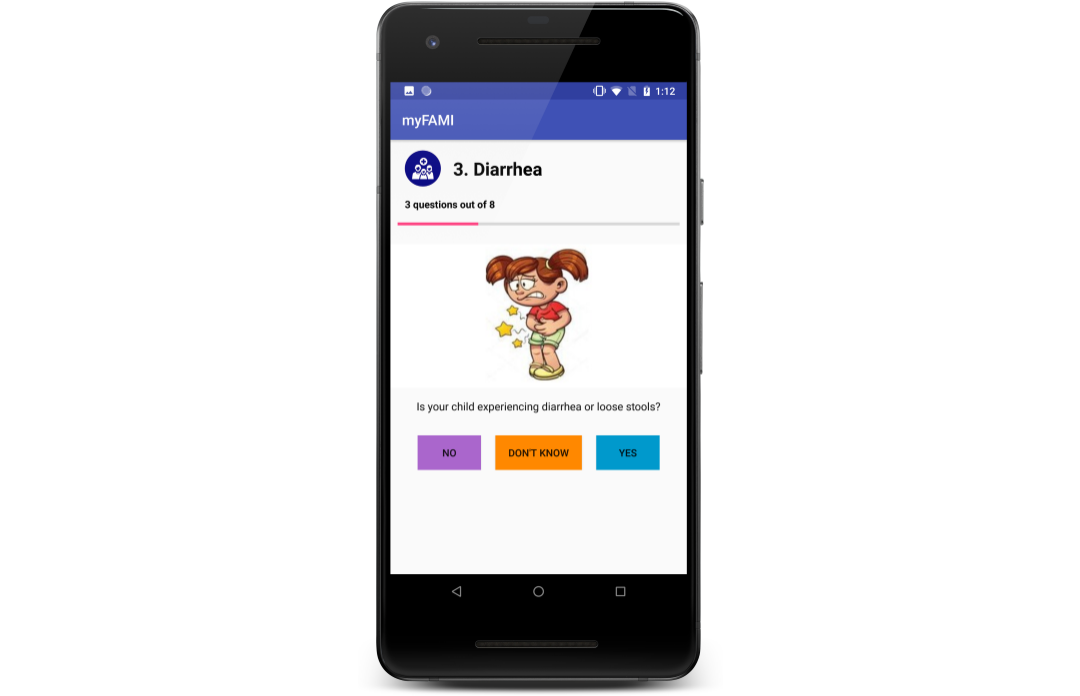
myFAMI app screenshot displaying one of the symptom-based survey questions.

**Figure 3:**
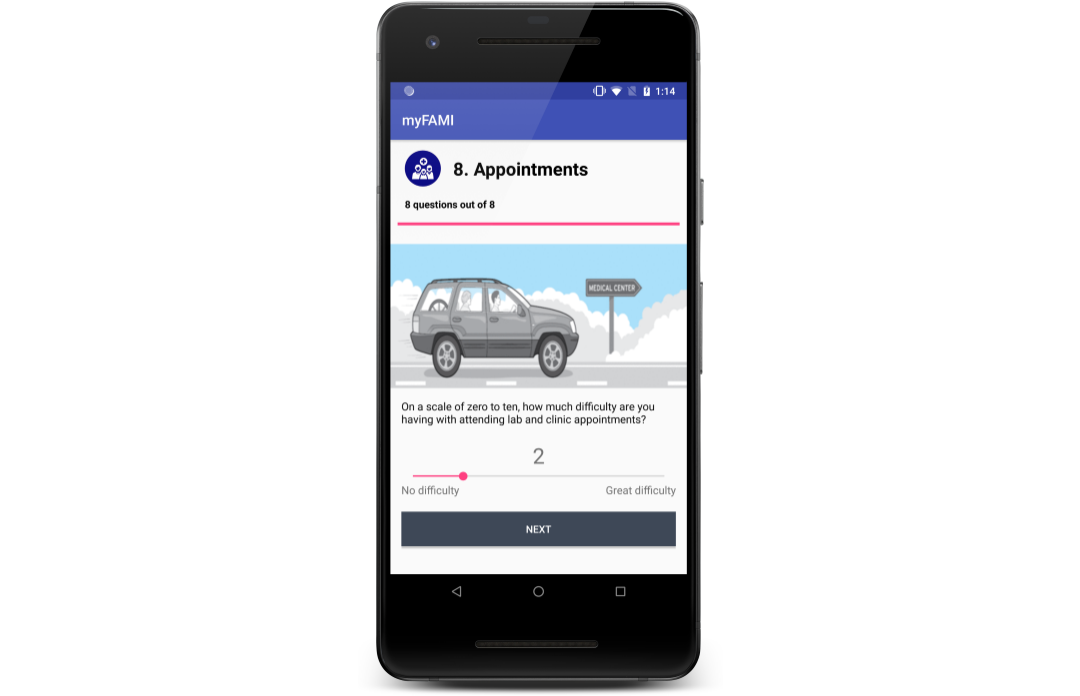
App screenshot displaying one of the self-management–based survey questions.

The mHealth intervention, myFAMI, included the use of a smartphone app and nurse response (video or telephone) to support family self-management for family members after their child’s transplantation. myFAMI promoted daily communication initiated by an in-app notification and completed by the participating family member for 30 days after discharge. Specifically, family members received an in-app notification at 8:00 AM reminding them to answer the 8 daily questions within 2 hours (ie, by 10 AM); 5 of the 8 questions were symptom-based (fever, vomiting, diarrhea, pain, other illness) and 3 were self-management–based (coping, medication administration, and medical appointments). Preidentified triggers for each question resulted in an alert to the research nurse who subsequently contacted the family to discuss any of the symptom(s) and self-management issues within 2 hours of receiving the alert. The preidentified triggers were defined as follows: (1) an answer of “yes” or “don’t know” for the 5 symptom-based questions and (2) an answer of 3 or greater using a scale of “0” (no difficulty) to “10” (great difficulty) for the self-management questions. Figure 1 provides a summary of the app workflow and Figures 2 and 3 illustrate 2 of the 8 survey question pages [27]. Further details regarding myFAMI, study workflow, and app screenshots are available in our protocol manuscript [28] and app development manuscript [27].

## Methods

### Design

This study involves an evaluation of the acceptability of an mHealth intervention (myFAMI) as experienced by the intervention family members participating in a randomized controlled trial comparing myFAMI with standard postdischarge follow-up care [24]. The main pilot study was a randomized controlled trial that evaluated the efficacy of myFAMI [24]. The data for this report include qualitative data from family members who reported their experiences of participating in the mHealth intervention to discern strengths and uncover opportunities for improving the acceptability and outcomes of myFAMI.

### Participants

A convenience sample of family units of pediatric heart, kidney, and liver transplantation recipients was enrolled. For this report, a family unit is defined as one of the following: (1) a primary family member only or (2) a primary and secondary family member. This may include a variety of family unit combinations such as mother/father, mother/aunt, father/grandmother, mother/grandmother. Over a 17-month period, family units were enrolled at 4 major pediatric transplantation programs in the United States. Participants were deemed eligible to participate if they were (1) 18 years of age or older; (2) English speaking; and (3) had a child (<18 years of age) who received a heart, kidney, or liver transplantation with expected discharge to home from the hospital [24]. Likewise, family members were deemed ineligible to participate (1) if they had communication or cognitive impairment that limited their ability to complete the myFAMI questionnaires or (2) if the child had a prior transplantation that would minimize the potential for an experiential effect. A family unit was *not* excluded if only 1 family member (primary family member) was interested in participation.

### Procedure

After receiving institutional review board approval at each of the 4 study sites, eligible family members and their child were identified and approached for voluntary participation and informed consent/assent was obtained. On the day of hospital discharge, family units were randomized to the control or intervention (myFAMI) group.

The qualitative data used for this analysis were provided by family members who were assigned to the myFAMI app and participated in a 30-day postdischarge telephone interview. One component of the telephone interview consisted of open-ended questions focused on the overall experience of using the app (Table 1). Overall, 5-10 minutes was devoted to this qualitative component for the myFAMI group. To ensure consistency of interview questions, interviews were conducted by a single interviewer from the Pediatric Translational Research Unit at the main study site using a semistructured interview guide. The interviews were audio-recorded and transcribed by the single interviewer who also verified the accuracy of the transcription and corrected as needed.

**Table 1:** Qualitative questions in the 30-day follow-up interview.

Description	Questions
Challenges	Tell me about any challenges you had with using the app? Technical challenges with the app itself maybe something we can improve for future use.
Functionality	Tell me if you feel the app helped you after discharge? Helped you to manage your child at home? How much time did it take you to complete the app every day? Did the time spent with the app seem too long, too short, or just right?
Overall experience	Can you tell me more about your experience with using the app? Did you think it was easy or hard to use? Why?
Use of the app	Did you answer the app every day for 30 days? If not, what were some of the reasons why you did not complete the app? (ie, technical problems with the app or app did not work, forgot that day)
Call experience	Did you receive a call from the research nurse? If yes, what went well? What did not go well?
Recommendations	What recommendations do you have for improving the app? Would you recommend using the app for other families?

### Data Analysis

This qualitative approach was guided by content analysis, a systematic and rigorous means of describing the family member experience [29]. The systematic coding of responses was an iterative process leading to emerging themes that portrayed each family’s perspectives on, acceptance of, and recommendations for myFAMI [29]. Two experienced qualitative researchers (SL and EF) independently reviewed the interview transcripts and coded data into categories of responses. The 2 reviewers then worked together to systematically compare the coded responses and develop initial themes. Through an iterative process, transcripts and themes were reevaluated to generate the final themes. Rigor was assured by use of an audit trail documenting development of final themes. Differences in coding in the independent review phase were resolved by discussion and consensus between the 2 reviewers.

### Ethics Approval

The study was approved by the Institutional Review Board at the Children’s Hospital of Wisconsin (IRB approval number 1183697) and at each individual enrolling transplantation center.

## Results

### Demographics

A total of 21 family units (primary family member only or primary and secondary family members of the transplanted child) had 30-day postdischarge interview data available for analysis. The final sample consisted of 32 primary and secondary family members. The majority of this sample comprised family members of children who received liver transplantations (13/21, 62%). An overwhelming majority of the children (20/21, 95%) and family members (30/32, 94%) were White. The child age spanned from infant to adolescent (range 30 days to 17 years). The age range for family members was 25-63 years. Additional demographic data are listed in Table 2.

**Table 2:** Demographics.

Characteristics			Values
Family member age (n=32), years, median (IQR)			36.5 (32-46)
**Family member gender (n=32), n (%)**			
	Female	20 (62)	
	Male	12 (38)	
**Family member race (n=32), n (%)**			
	White	30 (94)	
	Asian	2 (6)	
**Marital status (n=32), n (%)**			
	Married	24 (75)	
	Single	8 (25)	
**Relationship to child (n=32), n (%)**			
	Mother	18 (56)	
	Father	11 (34)	
	Grandmother	1 (3)	
	Other	2 (6)	
Child age (n=21), years, median (IQR)			8 (3-10.5)
**Child gender (n=21), n (%)**			
	Female	10 (48)	
	Male	11 (52)	
**Child race (n=21), n (%)**			
	White	20 (95)	
	Asian	1 (5)	
**Child transplantation type (n=21), n (%)**			
	Heart	6 (29)	
	Kidney	2 (10)	
	Liver	13 (61)	

### General Acceptance

In the 30-day follow-up interview, nearly all participants (31/32, 97%) reported that the app was easy to use. In discussing certain features, 41% of family members (13/32) specifically mentioned that design features made the app easy to use. Question setup and phrasing were mentioned as a good part of the app design (4/32, 13%). One participant mentioned liking the pictures that were added with the questions, while 3 participants stated the notifications/reminders to complete the survey were good features. For example, a participant stated, “It sent notifications; reminders were helpful; simple and easy to use”. Another parent stated, “Easy to use, being able to click yes or no”. When asked about time spent with the app, the majority of family members (29/32, 91%) said it took 2 minutes or less to complete the app daily, and that time spent in the app was “just right.”

Family members who did not complete the app every day reported various reasons for missing days, including that they forgot (2/32, 6%), were away from the child (1/32, 3%), or were not sure (2/32, 6%). One parent stated, “2 or 3 times I wasn’t able to answer, days I may have gone home and wasn’t next to him [child], didn’t want to provide inaccurate information”. One other family member stated, “I believe I missed one day, can’t remember why”.

When asked about their experience with the app, family members indicated high levels of general acceptance, with 88% (28/32) recommending use of the app for future candidates. Supporting the theme of acceptance, a family member reported, “Yes [they would recommend it to other families], great to have the psychological safety net”, while another reported that they “highly recommend [use of the app]”. One family member did not recommend the app without explanation and another said s/he would recommend the app clarifying that “would recommend if it [the app] was geared towards helping people understand symptoms of infection and rejection”.

### Positive Interactions

Overall, among the 32 participating primary and secondary family members, 163 triggers were generated based on answers provided in the app. When a trigger alert was generated, the family member received a call from the research nurse to discuss reason for the alert. The nurses responded to 99.3% (162/163) of the trigger alerts within 2 hours. In all, 23/32 (72%) family members who were interviewed reported receiving a call from the research nurse; 21/23 (91%) of these family members stated that the calls with the research nurse were positive interactions (Table 3) and that the interactions with the research nurse were helpful (5/23, 22%). One family member supported this by saying, “Helped with adjustments in the beginning; she [the nurse] was very nice”, while another family member stated, “Received quick calls and they were beneficial”. Others who responded complimented the research nurses beyond the point of stating that it was a helpful interaction, that is, “Very positive and thorough; very caring, genuinely helpful”. The family members who used a video call reported a positive experience and stated, “the Zoom interaction worked well; the ability to share real time was best”. When asked what did not go well with the research nurse, only 1 participant (4%) answered by stating “Nice if a ‘5’ would constitute a trigger versus a ‘3’”.

**Table 3:** Themes and exemplar family member quotes.

Theme/category	Family member response
General Acceptance	*Easy to use, multiple choice was helpful* *Reminders were handy* *Super easy and fast* *Easy, and I’m not that tech savvy* *Easy, straight forward, within minutes someone would call us; reassuring as we had problems with diarrhea secondary to potassium levels* *Easy to use, repetitive in nature helped with awareness*
Positive Interactions (with the research nurse)	*Interactions were great* *Direct calls with RNs went well* *Went well [conversation with nurse], friendly and open to conversation, beneficial* *Research nurses were good, positive interactions* *RNs were good and knowledgeable* *Everyone [nurses] were informative and supportive, positive experience* *Got better talking with nurses because of the app*
Home Management: Helped Manage After Discharge	*Yes, I can get a hold of nurse; helped me become less anxious* *I think it helped initially to focus in on potential rejection symptoms; helped reassure that someone would call and talk through* *The ‘difficulty’ questions weren’t particularly helpful, but didn’t hurt, might be valuable to some users* *Nice to know that is there was a problem they would call back right away* *Awareness versus management, didn’t take symptoms for granted* *It keeps you cognizant of what to look for* *Yes, couple of times we had difficulty with taking medications and nurse provided suggestions that helped* *The app helped to take inventory of what was going on* *If we had questions about the PICC line, the team was able to respond with direct feedback*
Opportunities for Improvement	*Good app, I liked it, possibly be able to open it back and do a follow-up note. It would be nice to have access to an ongoing record* *Maybe change your parameters, I thought a ‘3’ was a good score so why did it cause a trigger? After than I put a ‘2’ and didn’t get a call* *Continue beyond the 30-day mark*

### Home Management

The majority of participants (23/32, 72%) stated that the app allowed them to better manage their child’s medical-related care after hospital discharge. The family members noted that the app helped them to be aware of symptoms and monitor the child for rejection. They specifically identified the value of access to the nurse for managing problems and resolving difficulties with their child’s treatments. The app and response by the nurse provided support, reassurance, and help with managing emotions, as one participant stated, “if I put in an illness the nurse called, it helped me manage” and another participant stated, “it helped me focus on getting him [child] better”. As many as 5 participants (16%) reported that the app did not help in managing the child (ie, “not really” or “didn’t add much value”). Specifically, 1 family member stated, “Not really manage, more of an assist”.

### Opportunities for Improvement

Although there were high levels of general acceptance, 25% of participants (8/32) also reported challenges, most frequently (4/8) technology-related challenges. Specifically, 1 participant stated, “In the beginning, the app didn’t work well on the phone; a study team member helped to get it working”. Two other participants reported feeling uncertain while using the app. For example, 1 participant stated, “When it talked about bowel movements, I’d question whether I should say ‘yes’ or ‘no’; if it changed a little, I wasn’t sure how to record”. Two participants stated that their only challenge was remembering to do the daily questions. One family member said, “No challenges other than remembering to complete it at times” and another parent stated, “the biggest challenge was remembering to complete it”.

In considering potential challenges, themes emerged from participant responses regarding recommendations to improve the app. In this, a few themes regarding ideas for improvement developed. First, 5 of the 32 family members recommended having the questions presented in a different order each day and with varying pictures. One participant supported this theme by stating, “switch up some of the questions.” Second, 4 participants recommended adding a space for extended feedback within the daily survey, that is, “Suggest adding a comments option. More chances for feedback...”. Four others recommended having more flexibility in calls with nurses. For example, “... the ability to leave a time to call back”. Lastly, 2 participants indicated that extending the use of the survey beyond 30 days would be beneficial, making a suggestion for, “longer, maybe out to the three-month mark”.

Table 3 lists additional family member responses for each of the 4 main themes.

## Discussion

### Principal Findings

The use of mHealth is an innovative approach that complements medical management for supporting the transition from hospital to home-based family self-management for medically complex children. This study aimed to understand parent perspectives and acceptability of a family self-management intervention (myFAMI) for the first 30 days after the child’s heart, kidney, or liver transplantation. It was important to learn the family perspective on acceptability of myFAMI to more fully understand how to further improve this mHealth app to promote successful family self-management during this high-acuity time [30]. The themes emerging from family member responses to use of myFAMI provide insight into the usability and acceptability of a delivery model for postdischarge continuation of surveillance, support, and care management using an mHealth app combined with triggered nurse responses to support family self-management.

Family members reported overwhelmingly positive perceptions of acceptability of the app. The high acceptability for an mHealth smartphone intervention has been similarly reported by adult liver transplantation recipients who stated that an app would help with their transplantation recovery [14]. mHealth has also been reported as an accessible and effective way to provide medical care and psychosocial support to adolescent and young adult lung transplantation recipients, engaging them in their own health care [31]. Further, a study with a platform similar to the one used in this study utilized SMS text messaging with adolescent solid organ transplantation recipients. The high acceptance rate (68%) supported mHealth technology as an acceptable means of communicating with the clinical care team [32]. The widespread use of technology offers unique opportunities for the use of patient-nurse interfaces to efficiently support patients and families at home.

Family members reported appreciation for the positive interactions during direct conversations with the nurse (video or telephone) in response to triggers from the app, describing the nurses as knowledgeable and supportive. Adolescent heart, liver, and kidney transplantation recipients reported similar benefit to participating in an mHealth SMS text messaging intervention. More specifically, the adolescents reported appreciation for receiving SMS text messages and knowing someone is checking in as enhancing their health care experience [32]. Adult liver transplantation recipients prefer to use virtual video visits and SMS text message options to facilitate a fast response to questions especially early in the transplantation recovery process [14]. This immediate posttransplantation recovery phase was the focused time frame for myFAMI. Each family member received individual attention specific to the needs identified in his/her answers to the 8 daily questions. The importance of individualized care was also reported by lung transplantation recipients during the COVID-19 pandemic [31].

myFAMI is an individualized family self-management intervention that leveraged mHealth to facilitate timely and effective patient-nurse communication [24]. Family engagement is an important aspect of the posttransplantation recovery process and highlights the significance of developing and fostering a mutually beneficial partnership between families and the health care team [17]. Parents of transplantation recipients have reported the importance of seeking out information to participate in the medical discussion and decision making [8]. This supports how family members were engaged in the intervention by answering the daily questions in the app and talking to the nurse if a trigger alert was generated.

Families valued the support from medical experts in addition to their primary transplantation team. Parents of transplanted children reported that they were worried about complications, documented stress and worry in the first 3 weeks after hospital discharge, and indicated that support from the medical team had a positive influence on their ability to cope [9]. The additional layer of surveillance or monitoring was also positively received by family members in this study. Caregivers of adult lung transplantation recipients identified the need for and importance of additional education from the transplantation team [33]. Providing ongoing nursing support and education is an opportunity to promote effective family self-management in the home environment. The myFAMI nurses overwhelmingly provided ongoing support and education during their conversations with the family members in this study.

Recommendations for improvement to the app were functional and included extending the time frame for communication beyond 30 days following discharge. The 30-day time frame for this study was chosen based on hospital quality indicators for readmission [34,35]; however, family needs may not follow this specific schedule. Family members also suggested variability in the app, including presenting the questions in different order each day and using a variety of pictures to make the tool more engaging. An opportunity to schedule times to speak with the nurse may meet individual family member priorities including extended hours. With this qualitative design, distinct recommendations from the end user (family member) can be considered for future versions of myFAMI. Incorporation of enhancements can support an improved experience for family members utilizing myFAMI in the future.

Study limitations exist. The study questions focused on the experience with and acceptability of myFAMI and did not address other opportunities for support or identify other family member needs. Non-English speaking families were excluded in this pilot study and future research with this population is indicated. The racial breakdown of the myFAMI group was predominantly White, indicating a clear lack of racial diversity. Future research can target diverse populations to identify racial or ethnic disparities experienced after transplantation and unique opportunities to support family self-management [36,37].

A strength of this study was identifying the family experience for different family structures including mom/aunt, mom/dad, and mom/grandmother. While the sample size was small and limited robust statistical analyses, it was sufficient to reach saturation with commonality reported by family member experiences. However, future studies would benefit from larger samples sizes that would allow for differentiation of the unique perspectives of each type of family member.

### Conclusions

This is the first study to qualitatively explore family member perceptions on the acceptability of a family self-management intervention for family members of pediatric heart, kidney, or liver transplantation recipients. Family members value and appreciate ongoing support and education specifically in the first 30 days after their child’s hospital discharge. myFAMI allowed for the opportunity for families to feel connected to the medical team while in their home environment. A fully powered clinical trial to determine outcomes of myFAMI is indicated to extend the knowledge for use of mHealth to promote successful family self-management. The use of mHealth in a transitional care delivery model that also includes nurse-patient interaction initiated through the mHealth app may be an effective way to improve overall outcomes for solid organ transplantation patients and their families and a model to consider in other chronic illness populations.

## Abbreviations

mHealth: mobile health
